# Regulation of early signaling and gene expression in the α-particle and bystander response of IMR-90 human fibroblasts

**DOI:** 10.1186/1755-8794-3-31

**Published:** 2010-07-29

**Authors:** Shanaz A Ghandhi, Lihua Ming, Vladimir N Ivanov, Tom K Hei, Sally A Amundson

**Affiliations:** 1 Center for Radiological Research, Columbia University, VC11-215, 630 West 168th Street, New York, NY, 10032, USA

## Abstract

**Background:**

The existence of a radiation bystander effect, in which non-irradiated cells respond to signals from irradiated cells, is well established. To understand early signaling and gene regulation in bystander cells, we used a bio-informatics approach, measuring global gene expression at 30 minutes and signaling pathways between 30 minutes and 4 hours after exposure to α-particles in IMR-90 fibroblasts.

**Methods:**

We used whole human genome microarrays and real time quantitative PCR to measure and validate gene expression. Microarray analysis was done using BRB-Array Tools; pathway and ontology analyses were done using Ingenuity Pathway Analysis and PANTHER, respectively. We studied signaling in irradiated and bystander cells using immunoblotting and semi-quantitative image analysis.

**Results:**

Gene ontology suggested signal transduction and transcriptional regulation responding 30 minutes after treatment affected cell structure, motility and adhesion, and interleukin synthesis. We measured time-dependent expression of genes controlled by the NF-κB pathway; matrix metalloproteinases 1 and 3; chemokine ligands 2, 3 and 5 and interleukins 1β, 6 and 33. There was an increased response of this set of genes 30 minutes after treatment and another wave of induction at 4 hours. We investigated AKT-GSK3β signaling and found both AKT and GSK3β are hyper-phosphorylated 30 minutes after irradiation and this effect is maintained through 4 hours. In bystander cells, a similar response was seen with a delay of 30 minutes. We proposed a network model where the observed decrease in phosphorylation of β-catenin protein after GSK3β dependent inactivation can trigger target gene expression at later times after radiation exposure

**Conclusions:**

These results are the first to show that the radiation induced bystander signal induces a widespread gene expression response at 30 minutes after treatment and these changes are accompanied by modification of signaling proteins in the PI3K-AKT-GSK3β pathway.

## Background

Non-targeted effects could significantly enhance risks associated with exposure to low doses of ionizing radiation, which occurs in clinical and environmental contexts. It has been established that signals from irradiated cells travel through medium and cellular junctions to produce changes in gene expression [1,2], ROS production [3] and moderate damage to DNA in bystander cells as measured by micronucleus formation [4]. Although there is no direct epidemiological evidence for these risks in humans, the potential importance of bystander effects is highlighted by the recent demonstration of radiation bystander carcinogenesis in a mouse model [5]. In primary fibroblasts the major players transmitting and maintaining signals between cells after irradiation appear to be soluble growth factors, cytokines, reactive oxygen species and extracellular matrix proteins [6,7]. A wealth of information exists on cellular events that occur 4 hours and later, including studies on gene expression [1,2,8-10] cytokine production [11], γ-H2AX measurement of DNA damage [12] and chromosomal end-points [13,14] in directly irradiated cells and bystander cells. However, the events that precede these and other well-studied bystander effects on chromosomes [15] and DNA damage [13] are yet to be elucidated. In studies using γ-H2AX as a marker of radiation induced DNA double strand breaks, the response in bystander cells was observed within 20-30 minutes after treatment [16]. A recent study confirmed a burst in cellular ROS levels 30 minutes after irradiation, followed by a 1-2 hr window during which double strand break repair foci were induced in α-particle irradiated and bystander cells [17]. Other studies on signaling in bystanders have proposed that an early increase in production of reactive radicals [3] and TNFα [1] after irradiation can induce a cytokine cascade, which is consistent with the large number of signaling and stress response genes induced in this study.

In the present study, we focused on early responses to understand primary events that are more proximal to the bystander signal. 30 minutes after exposure, both irradiated and bystander cells showed a burst of gene expression changes. Gene ontology and pathway analyses of differentially expressed genes at 30 minutes after treatment suggested responses that affect cell structure and motility, signal transduction, transcriptional regulation and cell-to-cell communication. We validated the microarray results by quantitative real-time PCR and found that there was good concordance between these two methods. We were also interested in time-dependent patterns of gene expression and focused our studies on genes that showed induction at 30 minutes in both irradiated and bystander populations. The selected genes encode proteins that are transcriptional targets of NF-κB, and time course analysis of mRNA levels further supported our previous suggestion [2,3] that this signaling pathway is activated in bystanders in a synchronized manner from 30 minutes onward. From our earlier study that focused on the 4-hour transcriptional response [2], we predicted the involvement of β-catenin activation in gene expression in irradiated cells. We have now investigated protein modifications in the AKT-GSK3β signaling pathway upstream of β-catenin transcriptional activation. Our results show that the radiation signal can trigger a cascade of changes in AKT-GSK3β-βcatenin pathway almost concomitantly with a widespread gene expression response as early as 30 minutes after exposure.

## Methods

### Cell culture, irradiation and RNA isolation

Early passage (population doubling <35) IMR-90 human lung fibroblasts (Coriell repository, NJ) were sub-cultured in Dulbecco's modified Eagle's medium (Gibco) and Ham's F10 medium in a 1:1 mixture plus 15% fetal bovine serum. Mylar-bottomed culture dishes were prepared as described previously [1]. An inner dish with a base of 38-micron-thick Mylar strips was inserted into a larger dish with a 6-micron Mylar base. The 38-micron Mylar completely shields the alpha particles so that only cells on the thinner Mylar areas of the dish were directly irradiated. Cells seeded in these dishes formed a contiguous layer. Cells were exposed to 0 (sham irradiated) or 50 cGy ^4^He ions (125 keV per micron) as simulated alpha particles using the track segment mode of the 5.5-MV Singletron accelerator at the Radiological Research Accelerator Facility of Columbia University. This dose corresponds to an average fluence of approximately 6-12 alpha particles per cell in the irradiated sections of the dish and the probability of a cell not receiving a particle is less than 0.25%. Four independent experiments were conducted.

Directly irradiated (outer dish) and bystander (inner dish) cells were separated at specified times after irradiation and RNA was isolated using Ribopure (Ambion, Life Technologies). RNA concentrations were measured using a NanoDrop ND-1000 spectrophotometer (Thermo Scientific) and RNA quality was monitored with the Agilent 2100 Bioanalyzer (Agilent Technologies, Santa Clara, CA). All RNA samples had RNA integrity numbers >9.0 [18] and 260 nm/280 nm absorbance ratios >2.

### Protein isolation and Western blot procedure

Directly irradiated (outer dish) and bystander (inner dish) cells were separated at specified times (30 minutes, 1 hour and 4 hours) after irradiation and trypsinized. For whole cell lysates, cells were collected, washed and lysed in 25% glycerol, 40 mM HEPES at pH 7.5, 1 mM DTT, 0.35 M NaCl, 0.5% NP-40 and Protease inhibitor mixture (HALT, Thermo Scientific). Separation of nuclear and cytoplasmic fractions was performed as recommended in the manufacturer's protocol for NucBuster™ from EMD Biosciences (Darmstadt, Germany). Protein concentrations were determined using the bicinchoninic acid method (Pierce) and measured using the Nanodrop-1000 spectrophotometer (Thermo Scientific). 50 micrograms of protein was used for western analysis and separated on 10% polyacrylamide gels. Primary antibodies were from Cell Signaling Technology, Boston: anti-AKT (cat# 9272), anti-phospho-AKT (S473) (cat# 9271), anti-GSK3β (cat# 9315), anti-phospho-GSK3β (S9) (cat #9336), anti-β-catenin (cat# 9562) and anti-phospho-β-catenin (S33/37/T41) (cat# 9561). Other antibodies were purchased from Millipore: anti-actin (cat# MAB1501) and Sigma: anti-TBP (cat# T1827). Secondary antibodies were conjugated to horseradish peroxidase and signals were detected using enhanced chemi-luminescence (Amersham, GE). Relevant bands were quantified by densitometry using Image J, background corrected and normalized to actin levels, then compared to time matched controls.

### Microarray Hybridization and Analysis

RNA isolations were performed in parallel across irradiated, bystander and sham-irradiated samples, so that all samples were collected from one sub-cultivated pool of IMR-90 cells that were seeded from a single cryo-vial. After treatment, cells were lysed in pools from Mylar dishes at 30-minute, 1-hour, 2-hour, 4-hour, 6-hour and 24-hour time points. We repeated the experiment four times to provide four biological replicates. We analyzed the 30-minute RNA pools by microarray hybridization. Cyanine-3 (Cy3) labeled cRNA was prepared from 0.3 μg RNA using the One-Color Low RNA Input Linear Amplification PLUS kit (Agilent). Dye incorporation and cRNA yield were monitored with the NanoDrop ND-1000 Spectrophotometer (Thermo Scientific). 1.5 μg of cRNA (>9 pmol Cy3 per μg cRNA) was fragmented, hybridized to Agilent Whole Human Genome Oligo Microarrays (G4112F) using the Gene Expression Hybridization Kit, and washed following Agilent's recommendations. Slides were scanned with the Agilent DNA Microarray Scanner (G2505B). Default parameters of Feature Extraction Software 9.1 (Agilent) and grid version 014850_D_F_20090416 were used for image analysis, data extraction, background correction, and flagging of non-uniform features.

Background corrected intensities were log_2 _transformed and median-normalized in BRB-Array Tools, Version 3.8.0 [19]. Non-uniform outliers or features not significantly above background intensity in 40% or more of the hybridizations were filtered out, leaving 27,576 features. A further filter requiring a minimum 1.5-fold change in at least 20% of the hybridizations was then applied yielding a final set of 6911 features that were used for subsequent analyses. The microarray data are available through the Gene Expression Omnibus database using accession number GSE18760.

BRB-Array Tools was used to identify genes that were differentially expressed between controls and directly or bystander irradiated cells using a random-variance paired t-test, an improvement over the standard t-test that permits sharing information among genes about within-class variation without assuming that all genes have the same variance [20]. The test compares the differences in mean log-intensities between classes relative to the expected variation in mean differences computed from the independent samples. Genes with p-values less than 0.005 were considered statistically significant. The false discovery rate (FDR) was also estimated for each gene using the method of Benjamini and Hochberg [21], to control for false positives.

### Quantitative Real-Time PCR (qRT-PCR)

The High-Capacity cDNA Archive Kit (Life Technologies, Foster City, CA) was used to prepare cDNA from total RNA. A custom low-density TaqMan array (Life Technologies, Foster City, CA) was designed using validated assays (additional file 1). Genes for inclusion on the low-density array (LDA) were selected on the basis of differential expression and low FDR, and seven previously selected endogenous control genes [2] were also included. For gene validation studies, 100 ng cDNA was used as input for LDAs. Quantitative real time PCR reactions were performed with the ABI 7900 Real Time PCR System using Universal PCR Master Mix (Life Technologies), with initial activation at 50°C for 120 seconds and 94.5°C for 10 minutes followed by 40 cycles of 97°C for 30 seconds and 59.7°C for 60 seconds.

Relative fold-inductions were calculated by the ΔΔC_T _method as previously used [22] using SDS version 2.3 software (Life Technologies). We applied geNorm [23] to the seven endogenous control genes on the LDAs to determine the most appropriate genes for normalizing the results. The LDA data was normalized to the geometric mean of peptidylprolyl isomerase A (*PPIA*) and ubiquitin C (*UBC*) gene expression levels.

### Gene ontology and pathway analysis

The genes responding significantly (p < 0.005) to either direct alpha particle or bystander irradiation were imported into DAVID, the database for annotation, visualization and integrated discovery http://david.abcc.ncifcrf.gov/[24]. The genes/proteins in our list were mapped to DAVID identifiers, and then functionally annotated using the DAVID biological processes and molecular function categories. The number of genes in each functional classification category was compared against the number of genes from the NCBI human genome in that category. The one-tailed Fisher exact t-test probability value was used to statistically determine over- or under- representation of classification categories, Bonferroni corrected p-values less than 0.05 were considered significant.

The sets of genes significantly responding to direct or bystander irradiation (p < 0.005) were also imported into Ingenuity Pathways Analysis (IPA) (Ingenuity^® ^Systems, http://www.ingenuity.com) to analyze network interactions between the genes. The imported genes were mapped onto a global molecular network developed from information contained in the Ingenuity Pathways Knowledge Base. Networks of these significantly responding genes were then algorithmically generated based on their connectivity. The biological functions that were most significant to these networks were determined, and Fischer's exact test was used to calculate p-values determining the probability that each biological function assigned to a network was due to chance alone. We also identified the IPA canonical pathways that were most significant within the differentially expressed gene sets.

## Results

### Early gene expression in irradiated and bystander cells

In four independent experiments, RNA was extracted from control, directly irradiated, and bystander IMR-90 fibroblasts 30 minutes after exposure and was hybridized to Agilent Human whole genome arrays. Using the class comparison tool of BRB-Array Tools [19], we identified genes with significantly different expression compared with controls. In directly irradiated cells, 709 genes were differentially expressed (p < 0.005 and false discovery rate (FDR) < 5%; additional file 2). In bystander cells 407 genes were differentially expressed (p < 0.005 and FDR < 8.5%; additional file 3). 293 genes responded significantly to both direct and bystander irradiation.

### Gene ontology analysis

We then analyzed the differentially expressed gene lists from the microarray studies for enrichment of gene groups using the DAVID database [24]. The first step in this analysis was to map gene symbols to DAVID identifiers. In bystander cells, 292 DAVID identifiers were mapped from the list of 407 differentially expressed genes, and in the directly irradiated cells, 516 out of 709 differentially expressed genes mapped to DAVID identifiers. The most significant biological processes indicated in bystander cells were in categories related to protein modification (p-value 7.6 × 10^-4^) and cell surface receptor mediated signal transduction (p-value 9.2 × 10^-3^) (Table 1). Biological processes that were significant in the bystander response: protein modification, cell surface receptor mediated signal transduction, cell structure and motility, cation transport and ion transport; were also significant in α-particle irradiated samples. The most significant category of differentially expressed genes in the irradiated condition was cation transport (p-value 6.2 × 10^-8^), which was also significant in the bystanders (p-value 2.0 × 10^-2^). Other significant categories in the directly irradiated cells, such as G-protein mediated signaling (p-value 8.5 × 10^-4^) and cell surface receptor mediated signal transduction (p-value 4.4 × 10^-3^) suggest a considerable involvement of activity at the cell membrane and inter-cellular signaling in the early response of directly irradiated cells, while the early response in bystanders was dominated by effects on signal transduction and cellular structure.

**Table 1:** Gene ontology analysis on biological processes

PANTHER Biological Processes	BYSTANDER	IRRADIATED
BP00063:Protein modification	7.63 × 10^-4^	2.24 × 10^-2^
BP00103:Cell surface receptor mediated signal transduction	9.22 × 10^-3^	4.38 × 10^-3^
BP00285:Cell structure and motility	1.05 × 10^-2^	2.98 × 10^-4^
BP00143:Cation transport	2.00 × 10^-2^	6.16 × 10^-8^
BP00142:Ion transport	2.00 × 10^-2^	1.51 × 10^-2^
BP00044:mRNA transcription regulation	NS	5.66 × 10^-7^
BP00071:Proteolysis	NS	6.18 × 10^-4^
BP00104:G-protein mediated signaling	NS	8.51 × 10^-4^
BP00020:Fatty acid metabolism	NS	2.32 × 10^-3^
BP00067:Protein glycosylation	NS	1.92 × 10^-2^
BP00292:Other carbon metabolism	NS	2.66 × 10^-2^
BP00102:Signal transduction	NS	2.84 × 10^-2^
BP00040:mRNA transcription	NS	2.88 × 10^-2^
BP00064:Protein phosphorylation	NS	3.00 × 10^-2^
BP00124:Cell adhesion	NS	4.60 × 10^-2^

To identify significant PANTHER biological processes categories, p-values are Bonferroni corrected. NS is not significant (p > 0.05).

Examination of the molecular functions of significantly changed genes (Table 2) indicates that most of the early changes in gene expression in irradiated cells are related to cytoskeletal functions involving actin binding cytoskeletal protein (p-value 2.1 × 10^-4^), suggesting that irradiation results in a rapid rearrangement of cell structure components. In bystanders, the most significantly affected molecular functions were non-receptor serine/threonine protein kinases enzymes (p-value 8.2 × 10^-3^) and cytoskeletal proteins (p-value 4.6 × 10^-2^). As with biological processes, no significantly responding molecular functions at 30 minutes were unique among the bystanders.

**Table 2:** Gene ontology analysis on molecular functions.

PANTHER Molecular Functions	BYSTANDER	IRRADIATED
MF00213:Non-receptor serine/threonine protein kinase	8.20 × 10^-3^	2.39 × 10^-2^
MF00261:Actin binding cytoskeletal protein	4.62 × 10^-2^	2.05 × 10^-4^
MF00231:Microtubule binding motor protein	NS	3.23 × 10^-3^
MF00091:Cytoskeletal protein	NS	3.62 × 10^-3^
MF00099:Small GTPase	NS	4.49 × 10^-3^
MF00264:Microtubule family cytoskeletal protein	NS	2.70 × 10^-2^
MF00224:KRAB box transcription factor	NS	4.08 × 10^-2^
MF00262:Non-motor actin binding protein	NS	4.10 × 10^-2^

To identify significant PANTHER molecular function categories, p-values are Bonferroni corrected. NS is not significant (p > 0.05).

### Quantitative real time RT-PCR validation of gene expression and time course analysis

**Figure 1:**
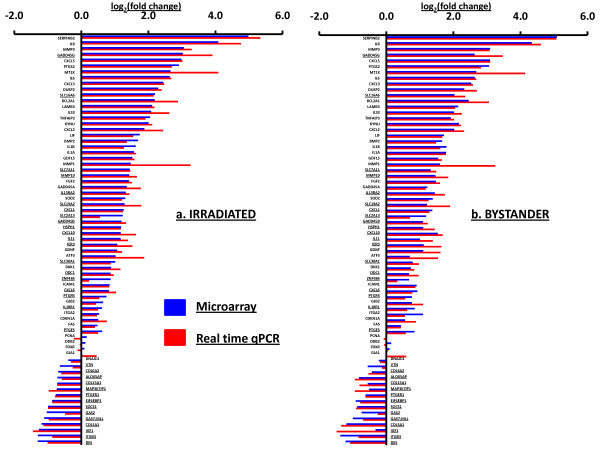
**Comparison of gene expression by microarray and qRT-PCR**. Relative gene expression is plotted as log_2 _(fold change) compared to matched sham-irradiated controls for irradiated (a) and bystander (b) samples at 0.5 hour after treatment. Each histogram is the mean of four biological replicates. Underlined genes are from class comparison results at 0.5 hour, the remaining genes were selected in our previous study [2] as responding at 4 hours. Microarray measurements (blue bars) and qRT-PCR measurements (red bars) are log 2 transformed ratios to controls; both up-regulated (right of the y-axis) and down-regulated genes (left of the y-axis) are shown.

We selected 34 genes that were differentially expressed in both direct and bystander samples for validation using Taqman real-time polymerase chain reaction (qRT-PCR). We also performed qRT-PCR on the 30-minute samples using 37 genes previously shown to change significantly at 4 hours [2], many (but not all) of which also responded in the 30-minute microarray data. The agreement between the two experimental approaches is very close, with a few exceptions where fold changes measured by qRT-PCR are higher than in microarrays (Figure 1). This effect has been observed previously by ourselves and others, and has been attributed to differences in probe choice and sequence specificity [25,26]. Some of the p53-regulated genes from the 4-hour responding set, such as *CDKN1A, GADD45A, FDXR, DDB2 *and *FAS*, were relatively unchanged by both measurement approaches at the early 30-minute time point, suggesting a delay in activation of the p53 pathway compared the cytokine/signal transduction effect in these cells [27]. In general, qRT-PCR analysis confirmed the up-regulation and down-regulation of a large number of mRNA at 30 minutes after treatment (Figure 1, additional files 2 and 3).

**Figure 2:**
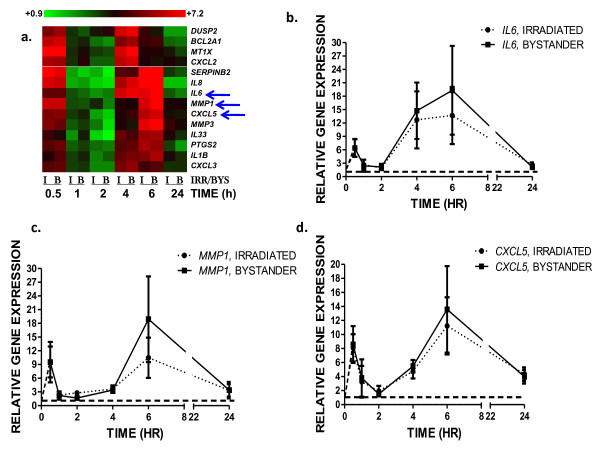
**Time course of gene expression after direct and bystander irradiation**. (a) Heatmap showing mean relative gene expression of genes with characteristic biphasic expression, irradiated (I) and bystander (B) ratios are shown adjacently over the time course. On the scale bar, red indicates up-regulation and green, down-regulation of mRNA compared to controls. qRT-PCR was used to monitor expression of *IL6 *(b), *MMP1 *(c) and *CXCL5 *(d), at 30 minutes, 1 hour, 2 hours, 4 hours, 6 hours and 24 hours after direct irradiation (closed circles) and bystander (closed square) exposure of IMR-90 cells. Gene expression was normalized to *PPIA *and *UBC *mRNA levels and is relative to expression in time-matched controls (dashed black line). Points are the mean and standard error of four independent experiments.

We used qRT-PCR to monitor the time dependent response of a subset of the genes that were induced early in both irradiated and bystander cells. Responses between 30 minutes and 24 hours were measured for NF-κB responsive genes, such as matrix metalloproteinases 1 and 3 (*MMP1 *and *MMP3*), chemokine ligands 2, 3 and 5 (*CXCL2, CXCL3 *and *CXCL5*) and interleukins 1β, 6 and 33 (*IL1B, IL6 *and *IL33*). The results are summarized as a heatmap (Figure 2a), which clearly shows the biphasic pattern of expression in both irradiated and bystander samples. We show transcriptional profiles of three individual genes to illustrate this pattern of expression and synchronization of the gene response. Expression of *IL6, MMP1 *and *CXCL5 *(Figures 2b, c and 2d) in bystanders was nearly identical to that in irradiated cells suggesting that these NF-κB responsive genes were subject to very rapid and synchronous activation.

### Pathway analysis

**Figure 3:**
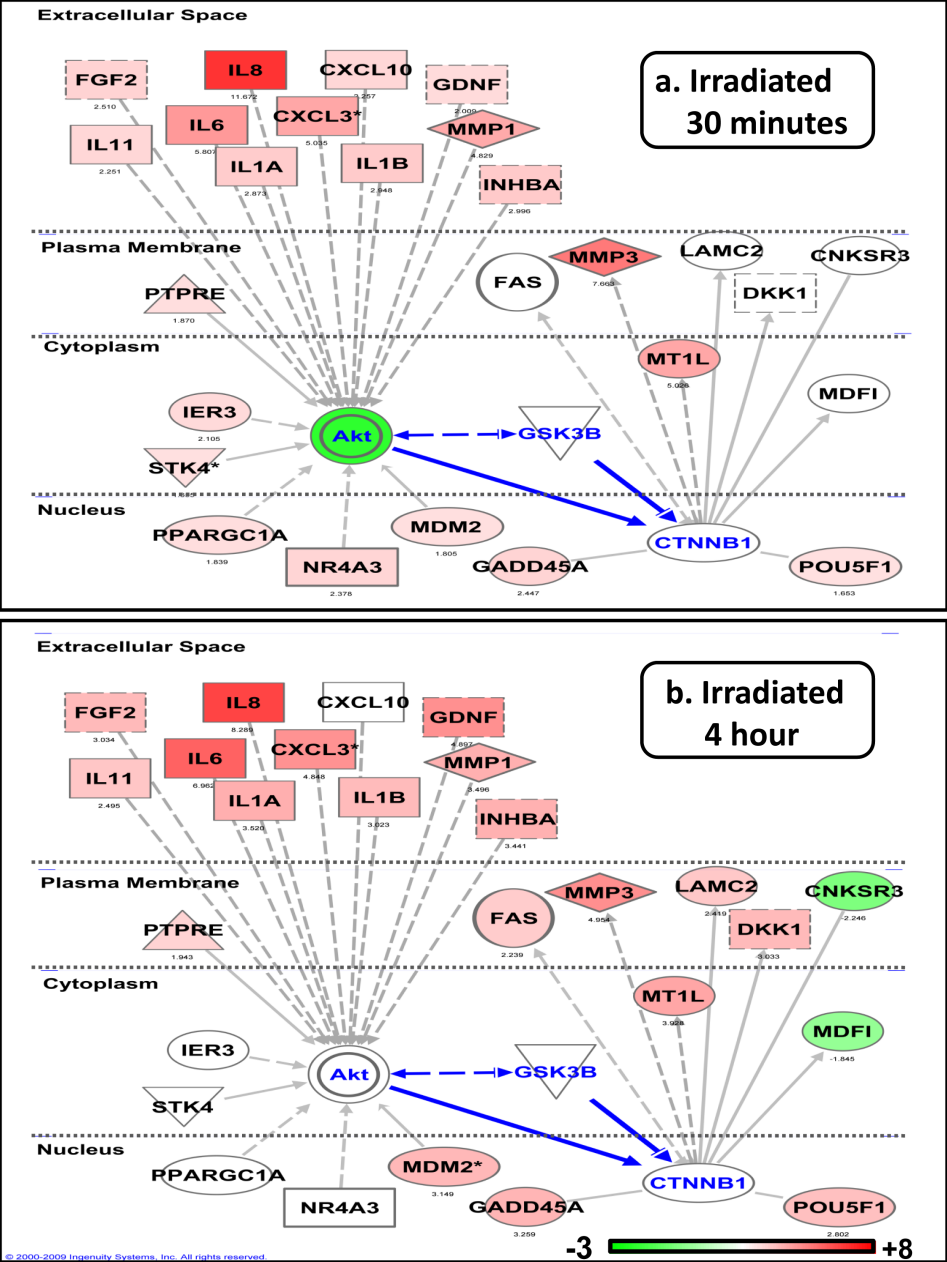
**Network analysis**. Ingenuity Pathways analysis (IPA) was used to generate the network overlaid with relative gene expression levels measured in directly irradiated cells at 30 minutes (a) and 4 hours (b). The network was built by selecting genes encoding inter-cellular signals responding early to radiation that could lead to activation of Akt. We hypothesized that Akt activation followed by post-translation modification of GSK3β and β-catenin would have an effect on gene expression. *FAS, DKK1 *and *LAMC2 *were induced at 4 hours but not at 30 minutes. *MMP3 *(figure 2a, heatmap) and *MT1L *were induced at 30 minutes as well as 4 hours. The dynamics of Akt signaling at the protein level in the 30-minute to 4-hour time interval are further investigated in Figure 4. Nodes representing gene products are displayed by cellular localization (extracellular space, plasma membrane, cytoplasm or nucleus). Node color indicates up-regulated genes (red) and down-regulated genes (green); scale bar indicates the range of expression ratios. Edges (lines and arrows between nodes) represent direct (solid lines) and indirect (dashed lines) interactions between molecules as supported by information in the Ingenuity knowledge base. We indicate Akt, GSK3β and β-catenin (*CTNNB1*) and their relationship edges in blue to highlight the signaling axis focused on in the following figure 4. Node shapes represent functional classes of gene products; rectangles with solid lines for cytokines, rectangles with dotted lines for growth factors, triangles for phosphatases, concentric circles for groups or complexes, diamonds for enzymes, and ovals for transcriptional regulators or modulators.

We then imported the lists of 709 and 407 genes from irradiated and bystander samples, respectively, into Ingenuity Pathway Analysis (IPA) to visualize pathway interactions of the responding genes/proteins. Top interacting networks of radiation responsive genes were significantly enriched for molecular and cellular functions of cell growth and proliferation (p = 10^-12 ^to 10^-4^), cell death (p = 10^-12 ^to 10^-4^) and cellular movement (p = 10^-10 ^to 10^-4^). The top bystander networks were significantly enriched for cell death (p = 10^-13 ^to 10^-4^), cell movement (p = 10^-11 ^to 10^-4^) and cell-to-cell signaling (p = 10^-9 ^to 10^-4^), additional file 4. Pathway analysis of significant networks indicated that the NF-κB transcription factor has a prominent role early in the bystander response. We also used IPA to connect gene networks to predict new regulatory hubs. Comparison of the 30-minute responding genes with the 4-hour responding genes published previously [2], predicted a possible role for β-catenin protein as a transcriptional activator of gene expression at 4 hours. This pathway is modeled in Figure 3 using IPA and described in detail in the discussion. We reasoned that investigation of protein modifications in the β-catenin signaling pathway at early time points after treatment could help us to understand the fine tuning of the early response to radiation.

### Activation of AKT-GSK3β-β-catenin in bystander cells

**Figure 4:**
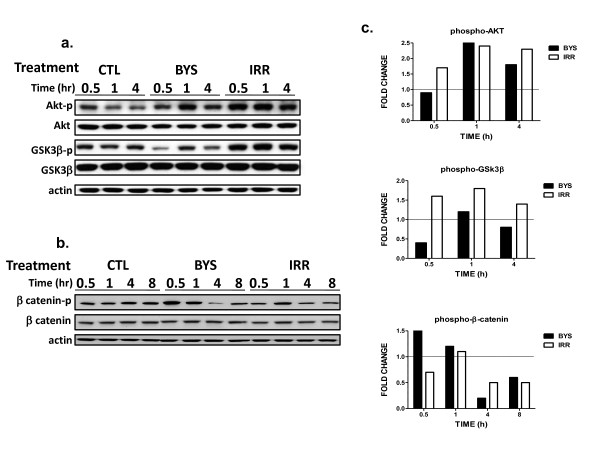
**Protein expression analysis of IMR-90 fibroblasts following irradiation**. Western blot analysis of un-irradiated control (CTL), bystander (BYS) and irradiated (IRR) cells comparing (a) phosphorylated-AKT, total AKT, phosphorylated-GSK3β, GSK3β and actin and (b) phosphorylated β-catenin, β-catenin and actin up to 8 hours. (c) Densitometric analyses were performed on representative immunoblots, bands were background corrected, normalized to actin and sham-irradiated controls. The relative levels of phosphorylated AKT, phosphorylated GSK3β and phosphorylated β-catenin are shown as fold change compared with controls. The thin black line represents time-matched controls.

We focused on the AKT-GSK3β-β-catenin axis of signaling in normal fibroblasts because pathway analysis predicted involvement of β-catenin as a transcriptional activator in the unfolding radiation bystander response. The results of western blot quantification of phosphorylated and basal protein levels of AKT kinase and GSK3β kinase are shown in Figure 4a, from 30 minutes to 4 hours after exposure. We observed an increase in the relative amount of activated AKT-P (Ser 473) in irradiated samples at 30 minutes, with consistent phosphorylation/activation of AKT through 4 hours post irradiation and a concomitant increase in GSK3β(Ser9) phosphorylation and inactivation. Bystanders appeared to lag behind the irradiated samples by an additional 30 minutes, showing increased levels of phosphorylated AKT in the 1 to 4 hour interval (Figure 4a and 4c). In parallel with this, GSK3β phosphorylation was increased at 1 hour in bystanders. Inactivated by phosphorylation, GSK3β can further alter the phosphorylation status of β-catenin and we measured cytoplasmic β-catenin and phosphorylated β-catenin levels (Figure 4b). Decreasing levels of phosphorylated cytoplasmic β-catenin could be the result of increased GSK3β phosphorylation in both irradiated and bystander cells. The observed decrease in β-catenin phosphorylation and the accumulation of β-catenin can lead to translocation to the nucleus where it may act as a transcriptional co-activator of gene expression of several genes such as *PTGS2 *and *FAS*.

## Discussion

In this study we focused on early events to better understand the early signaling that shapes the radiation bystander effect. Time dependent analyses of specific genes such as *PTGS2*, *IL8 *and *BCL2A1 *had previously shown dramatic biphasic changes with a large response as early as 30 minutes after exposure [2]. This suggested that the re-programming of gene transcription is initiated prior to 4 hours after exposure. We also wanted to investigate signaling pathways in order to identify candidates for the upstream initiating signals that may lead to the gene expression changes observed at later time points. Therefore, we investigated the early burst of gene expression, looking for potential signaling nodes using a bioinformatics approach in conjunction with more conventional analyses of protein modifications of transduction pathways.

We measured global transcriptional changes at 30 minutes after treatment and found even more differentially expressed genes (709 in irradiated cells and 407 in bystanders) than we had previously reported at 4 hours, when 197 genes were changed significantly in irradiated cells and 137 genes were changed significantly in bystander cells [2]. The general patterns of gene expression were confirmed by qRT-PCR. Many genes that showed increases in expression after 4 hours were also induced at the 30-minute time in both irradiated cells (127 genes) and bystanders (120 genes). In contrast to the prominent role of p53 previously observed in the 4-hour response, however, there was no such pronounced p53 response at 30 minutes (Figure 1a) in the irradiated cells.

Of the 709 genes affected in directly irradiated cells, 416 genes were not significantly altered in bystanders. Gene ontology analysis using DAVID indicated that the top functional categories of the direct-exposure-only genes were in mRNA transcription regulation and activation of gene expression. These functions were not significant in bystanders at early times indicating that irradiation rapidly induced processes that altered the transcriptional landscape within hit cells, in addition to the overall stress response characterized by the release of cytokines and inflammatory regulators. The early bystander response was associated with cytokine signaling, cell structure changes and extra-cellular communication, suggesting that bystander fibroblasts were responding by altering their own cellular environment (Table 1). We had already reported that the gene expression responses of cyclooxygenase2 (*PTGS2*), interleukin-8 (*IL8*) and BCL2 related protein A1 (*BCL2A1*) were comparable at 30 minutes and 4 hours after exposure [2]. These genes are known targets for regulation by the NF-κB transcription factor, and we predicted that the biphasic response in mRNA levels could be due to modulation by inhibitors of NF-κB[28]. In this study, we have verified that this was a frequent pattern of gene expression. Many genes that responded with an increase in mRNA levels at 4 hours showed a strong response at 30 minutes as well (Figures 1 and 2), suggesting co-ordinate regulation of genes with this biphasic pattern. These genes did not include canonical p53-response genes, which generally show peak induction at approximately 4 hours after irradiation, depending on the model system used. We further verified time dependent patterns of mRNA changes for 11 additional genes across the 24 hour time interval and found that they all showed peaks of induction after 30 minutes and 4 or 6 hours with a sharp decrease to control levels at the time between 1 and 2 hours, Figure 2a. This pattern of modular gene expression could be attributed to co-regulation of genes under the control of a transcription factor, such as NF-κB, acting as an early signal transducer [3,29].

We compared the two gene sets, 709 genes in irradiated and 407 genes from bystander, for significant biological functions in IPA, which categorizes genes by mechanism- and disease-related associations. Gene expression changes in both irradiated and bystander cells appeared to be relatively similar in top-scoring biological functions categories such as cancer, cellular growth, proliferation, and cell death, which are very broad descriptions for functions associated with regulation of cell numbers and tissue homeostasis (additional file 4). 301 cancer-related genes in irradiated cells gave a p-value range from 1.2 × 10^-17 ^to 5.08 × 10^-4 ^for the cancer functional category. For 199 differentially expressed genes from bystanders; the p-value range for the same category was 2.45 × 10^-16 ^to 2.36 × 10^-4^. This indicated similar enrichment of this biological response in both gene sets. However, a closer look at the individual genes in the two conditions revealed differences, which were expected because direct irradiation would cause cells to have a more pronounced DNA damage and cell cycle response. IPA grouped 70 genes from 407 bystander responding genes under the functional category "cell-to-cell signaling" In IPA these genes were sub-grouped further by specific biological activities related to cell-to-cell signaling. We ranked these sub-categories by decreasing order of number of genes affected and the highest ranked functions in bystander cells correlated with increases in activation, adhesion, communication, signaling, binding and stimulation of normal cells. From IPA and ontology analyses taken together, the mRNA changes suggest stimulation of cellular response in bystanders by signaling molecules as early as 30 minutes after treatment (additional file 4).

Pathway analysis of genes affected in irradiated cells after 4 hours suggested that in addition to p53 and NF-κB transcription factors, β-catenin (most probably in concert with LEF1) could be an important trans-activator of gene expression. We chose to focus on the AKT-GSK3β-β-catenin axis of signaling in normal fibroblasts because of its central role in response to growth factors, changes in the medium, cytokine signaling, cell structure changes and stress due to production of reactive radicals, all of which are components of the bystander response [30]. Previous studies on bystander cells after carbon ion or alpha particle irradiation have also implicated activation of Akt signaling based on up-regulation of target gene [9,31]. The activation of β-catenin as a nuclear activator of transcription is known to follow GSK3β phosphorylation by Wnt signaling or alternatively to be regulated through PKB/AKT activation [30,32]. Cells responded to irradiation by rapid activation of AKT via phosphorylation and concomitant inactivation by phosphorylation of GSK3β at 1 to 4 hours. In bystanders, there was a similar pattern of GSK3β inactivation, but with a lag of 30 minutes or more. Cytoplasmic levels of phosphorylated β-catenin decreased gradually, which is considered a good indicator of trans-location to the nucleus [32] and activation of gene expression. This effect was also observed in bystanders, so β-catenin activity as a transcriptional co-activator of gene expression may occur in both populations. We have summarized these findings in Figure 3, which is a proposed pathway model of AKT-GSK3β-β-catenin signaling pathway after irradiation. External signals such as growth factors, interleukins 6 and 8, which were induced early and secreted by irradiated cells could trigger activation of AKT in all cells [33] initiating the signaling cascade outlined in Figure 3, which results in gene expression activation of β-catenin target genes such as matrix metalloproteinase 3 (*MMP3*), Fas (*FAS*) and metallothionein 1L (*MT1L*). In irradiated cells at 4 hours, this transcriptional regulator potentially regulated expression of *MT1L *(+3.9 fold change), *DKK1 *(+3.0 fold change), *MMP3 *(+5.0 fold change), *LAMC2 *(+2.4 fold change) and *FAS *(+2.3 fold change) (Figure 3b). In bystander cells, only *MMP3 *(+4.0 fold change) and *MT1L *(+3.8 fold change) were detected as differentially expressed at 4 hours. Of the genes predicted to be β-catenin targets, only *MMP3 *showed the predicted NF-κB biphasic response that was similar in both irradiated and bystander cells. Although there is overlap and redundancy in the roles of both transcriptional regulators and target genes, our results suggest that this mechanism is active in both irradiated and bystander cells. The apparent lag between activation of this pathway in directly irradiated and bystander cells suggests that although it is clearly not the initiating signal, this pathway may play a role in the maintenance and later development of the bystander state. There is evidence for a lag in signaling between irradiated and bystander cells as observed by apoptosis induction in human fibroblasts [34], γH2AX foci induction after media transfer [35] and mutation induction in bystanders after treatment with conditioned media [36]. The mechanism driving the burst of cytokine and other gene expression seen in bystander cells at 30 minutes post-exposure has yet to be clearly elucidated.

We also observed some changes in AKT expression in our study. Although there was an overall increase in phosphorylation in both irradiated and bystander cells, total AKT protein levels were slightly decreased (Figure 4a). This could be a consequence of the significant down-regulation of *AKT2 *mRNA observed at 30 minutes (0.4 ± 0.1 fold change) (additional files 2 and 3) and 4 hours (0.5 ± 0.1 fold change) in both irradiated cells and bystanders. The AKT antibody we used detected all three isoforms of AKT, and the phospho-Ser473-AKT antibody recognized phosphorylation of Ser473 on AKT1 as well as the corresponding residues in AKT2 and AKT3. However, GSK3β is known to be a substrate for all AKT isoforms [37], and in our system inactivation of GSK3β could result from activation of all AKT isoforms. Further studies will be required to distinguish the role of decreased *AKT2 *mRNA specifically, and its contribution to phosphorylation and inactivation of GSK3β.

The importance of our study approach is that whole genome bio-informatics can help elucidate novel signaling networks that contribute to any phenotype. Exploring gene expression and signaling after irradiation led us to propose activation of an important signal transduction module, AKT signaling via β-catenin, as part of the cellular response in bystanders. Further verification of the role of β-catenin in bystanders will help us understand the complex nature of this response. In the field of radiation biology, there are few studies that use this approach, but a recent study on keratinocytes exposed to 1 cGy X-rays that identified the GATA3 transcriptional factor as a critical regulator of cellular radiation response at the genome level [38] is one example. Importantly, our study is the first to investigate the global transcriptional response within the first half hour after exposure with the goal of identifying signals more proximal to the generation of the bystander signal. This could have implications for the understanding of how cells respond *in vivo *and lead to understanding the rapid nature of the response.

## Conclusions

A rapid and widespread transcriptional response occurs following irradiation in human fibroblast cells, and is communicated rapidly to bystanders. We found that cell structure and cell-cell communication processes are triggered very quickly in irradiated cells and these changes precede cell cycle gene responses that occur at a later time. Timing is important because release of free radicals and cytokines occurs quickly after irradiation and identifying early signaling events is critical to a full understanding of the range and extent of changes in bystander cells. The wave-like response of many genes following radiation suggests coordinate control of a large number of genes especially those that are controlled by NFκB. Activation of AKT-GSK3β-β-catenin signaling early after irradiation and activation of target genes later in irradiated and bystander cells is a novel finding implicating a new pathway in bystander response. The connection between this signal transduction module, with the potential to converge extra-cellular communication with intra-cellular alterations of proteins and gene expression adds one more piece to the puzzle of stress response in a cell population where not all cells experience a direct ionization event.

## List of Abbreviations

ROS: reactive oxygen species; TNFα: tumor necrosis factor alpha; NF-κB: nuclear factor kappa-light-chain-enhancer of activated B cells; AKT: v-akt murine thymoma viral oncogene homolog; GSK3β: glycogen synthase kinase 3 beta; BRB: Biometric research branch; FDR: false discovery rate; qRT-PCR: quantitative real time Reverse Transcription-Polymerase Chain Reaction; DAVID: The database for annotation, visualization and integrated discovery.

## Competing interests

The authors declare that they have no competing interests.

## Authors' contributions

SAG participated in the design of the study, carried out the microarray analyses and real-time PCR, and wrote the manuscript. LM carried out the protein analysis. VI contributed to the protein analysis and the writing of this manuscript. TKH contributed to the writing of this manuscript. SAA conceived of the study, participated in its design and data analysis and helped to draft the manuscript. All authors read and approved the final manuscript.

## Pre-publication history

The pre-publication history for this paper can be accessed here:

http://www.biomedcentral.com/1755-8794/3/31/prepub

## Supplementary Material

Additional file 1 **PCR assay information**. Click here for file

Additional file 2 **Class comparison of genes differentially expressed 30 minutes after direct irradiation**. Click here for file

Additional file 3 **Class comparison of genes differentially expressed 30 minutes after bystander irradiation**. Click here for file

Additional file 4 **Comparison of pathway analysis in irradiated and bystander cells at 30 minutes after irradiation**. Click here for file
