# Supplementary material for: Regulation of early signaling and gene expression in the α-particle and bystander response of IMR-90 human fibroblasts
Source: BMC Med Genomics. 2010 Jul 29;3:31. doi: 10.1186/1755-8794-3-31 (PMC2919438; doi:10.1186/1755-8794-3-31)

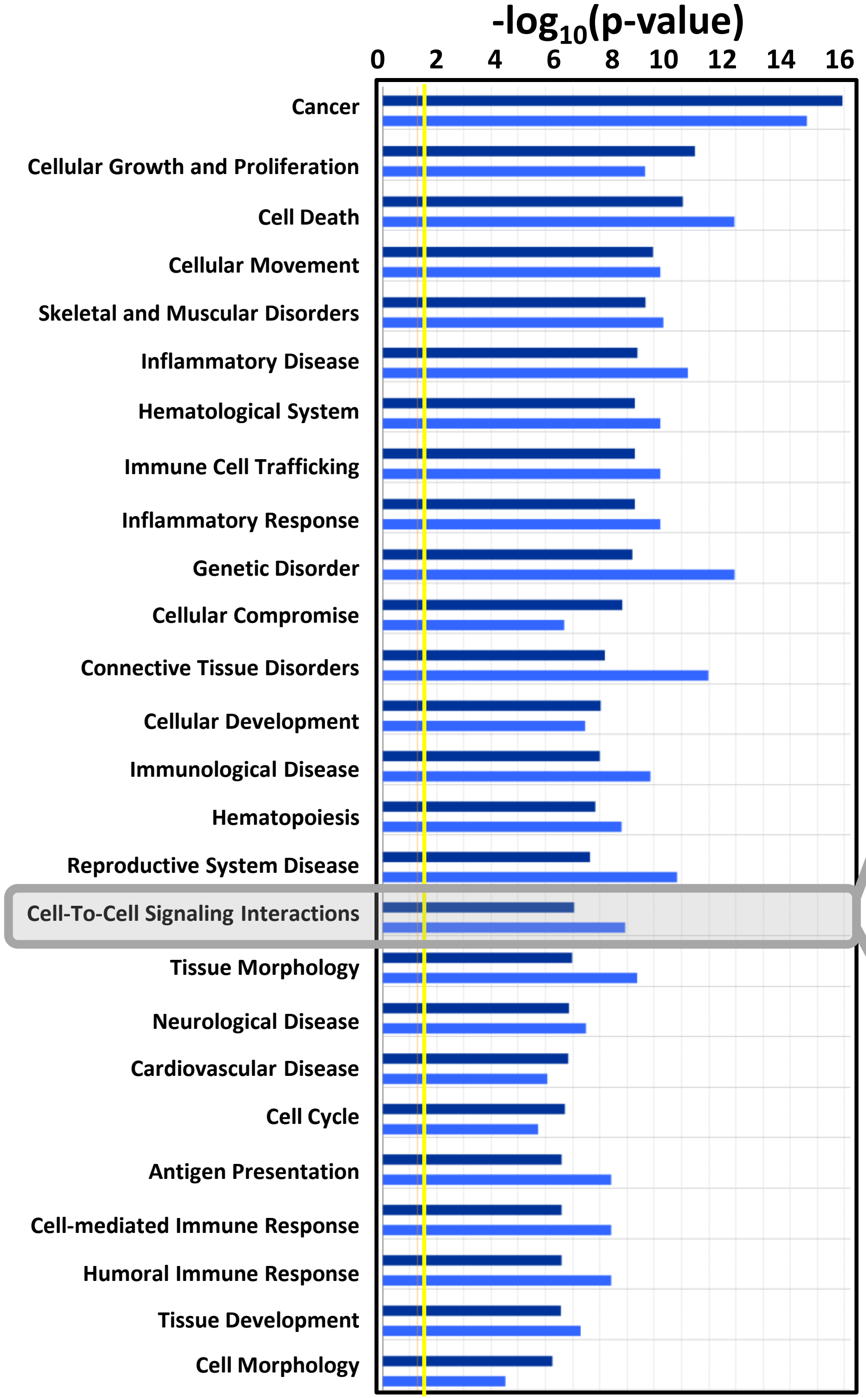

Additional file 4. Comparison of pathway analysis in irradiated and bystander cells A. IPA was used to compare two gene sets and display a score $-\log_{10}$(p-value), for inclusion of a subset of genes in a biological category. B. We illustrate the "cell-to-cell signaling" interactions gene subset and connected 70 genes changed in bystanders into a network. Arrows indicate direction of the relationship between molecules (nodes) and colors represent up-regulation (red) and down-regulation (green) of mRNA.

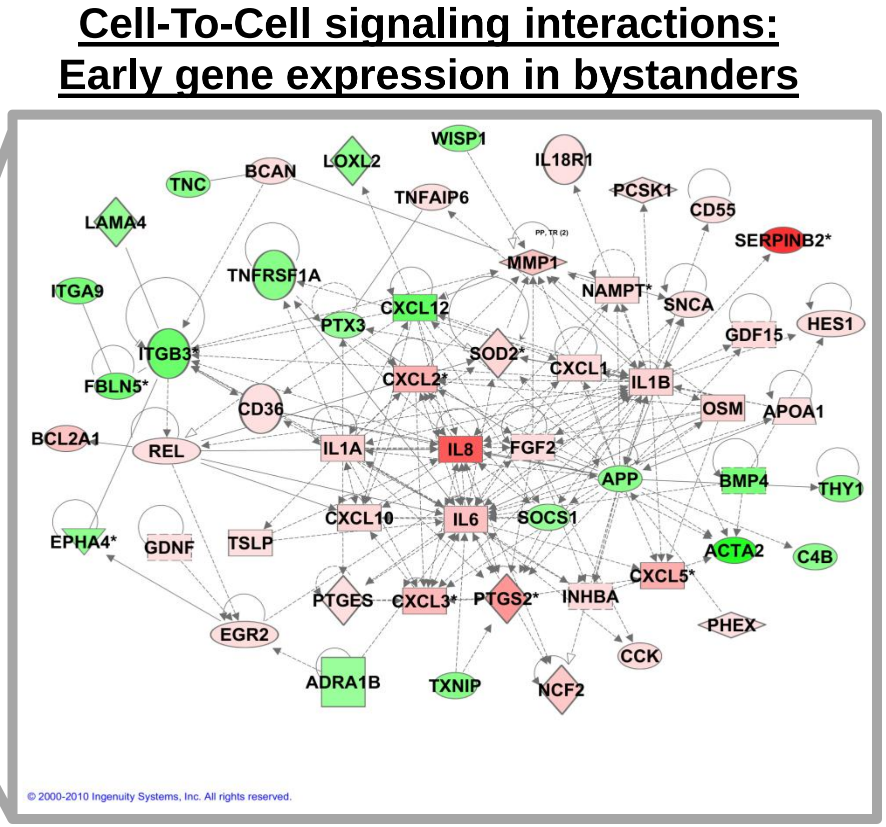

Supplement: Additional file 4 — Comparison of pathway analysis in irradiated and bystander cells at 30 minutes after irradiation. [file 1755-8794-3-31-S4.PDF]
